# Ruptured endometrioma in a nonpregnant patient: a case report

**DOI:** 10.1186/s13256-022-03361-3

**Published:** 2022-04-23

**Authors:** Hayley Young, Thanh-Lan Bui, Scott E. Cramer, Ryan O’Connell, Roozbeh Houshyar

**Affiliations:** 1 Department of Radiological Sciences, University of California Irvine, 101 The City Dr S Building 55, Box 140, Orange, CA 92868 USA; 2 Department of Pathology, University of California Irvine, 101 The City Dr S Building 1, Rm 3003, Orange, CA 92868 USA

**Keywords:** Ruptured endometrioma, Endometriosis, Hemoperitoneum, Computed tomography, Ultrasound, Case report, Abdominal imaging

## Abstract

**Background:**

Endometriomas are a type of ovarian cyst composed of degenerated blood products from hemorrhage of ectopic endometrial tissue. Endometriomas can rupture, causing hemoperitoneum, and present with signs and symptoms similar to other, more common abdominal emergencies. Therefore, they are not often diagnosed preoperatively. Ultrasound and cross-sectional imaging can assist in diagnosis of endometriomas. We present a case of ruptured endometrioma causing massive hemoperitoneum that was initially suspected to represent malignancy with carcinomatosis.

**Case presentation:**

A 32-year-old Hispanic woman presented with sharp abdominal pain and 15-pound unintentional weight loss over 6 months. Laboratory work was significant for a negative pregnancy test and elevated cancer antigen-125. Computed tomography of the abdomen and pelvis demonstrated a 13-cm complex cystic mass in the left adnexa with moderate hyperdense ascites and omental nodularity. Ultrasound demonstrated a large left adnexal complex cystic structure with internal echoes, and chest computed tomography showed no signs of intrathoracic neoplastic or infectious processes. Her presentation was concerning for malignancy with carcinomatosis. Fluid from a paracentesis was sent for culture and cytology. Diagnostic laparoscopy revealed that the left ovary had been completely replaced by an endometrioma, which had a small ruptured area superiorly. Brown deposits of endometriosis were present on the cyst, omentum, and various peritoneal linings. Tissue samples of the endometrium, myometrium, cervix, ovaries, fallopian tubes, peritoneum, omentum, and paracolic spaces were taken and showed no hyperplastic, dysplastic, or malignant cells on pathology.

**Conclusions:**

Ruptured endometrioma and ruptured hemorrhagic cyst should be included in the differential diagnosis when a premenopausal female presents with hemoperitoneum in combination with complex adnexal cystic masses in the absence of trauma. Cancer antigen-125 and cancer antigen 19-9 can be falsely elevated in the setting of ruptured endometrioma.

## Background

Endometriomas, a type of ovarian cyst, occur in 17–44% of women with endometriosis [1]. They are caused by hemorrhage of ectopic endometrial tissue within the ovary, resulting in degenerated blood products enveloped by ovarian parenchyma. Typically, endometriomas are composed of fibrotic walls with a lining of endometrial epithelium, stroma, and glands. While they are often managed conservatively, associated complications, such as rupture, may require surgical intervention. Rupture generally occurs in endometriomas larger than 6 cm, and affects less than 3% of known endometriomas [2]. However, a ruptured endometrioma may have serious consequences such as decreased fertility, adhesions, and pelvic pain [3].

According to current literature, ruptured endometriomas are rarely diagnosed preoperatively, as they share similarities with other, more prevalent abdominal emergencies [4]. In these instances, ultrasound and cross-sectional imaging may be able to assist in correctly identifying a ruptured endometrioma. Higher suspicion for a ruptured endometrioma will facilitate the decision to pursue a laparoscopy. Here we discuss a case of a ruptured endometrioma that presented with clinical and imaging findings that initially raised suspicion for ovarian neoplasm. We aim to clarify some of the key findings that are helpful for differentiating preoperatively between neoplasm and endometrioma.

## Case presentation

A 32-year-old Hispanic woman presented with sharp pain in her upper to mid abdomen. She had no significant medical history but reported 15-pound unintentional weight loss over the past 6 months. However, her abdomen grew in size during the same period. At time of admission, her abdomen was distended, and a 3/6 systolic murmur was heard at the left upper sternal border. Laboratory values were significant for leukocytosis to 17.5 × 10^3^/MCL (normal 4.0–10.5 × 10^3^/MCL), aspartate aminotransferase (AST) 273 U/L (normal 13–39 U/L), alanine transaminase (ALT) 149 U/L (normal 7–52 U/L), and cancer antigen-125 (CA-125) 4663 U/ML (normal < 35 U/ML). Urinalysis was positive for protein, ketones, and red blood cells.

**Fig. 1:**
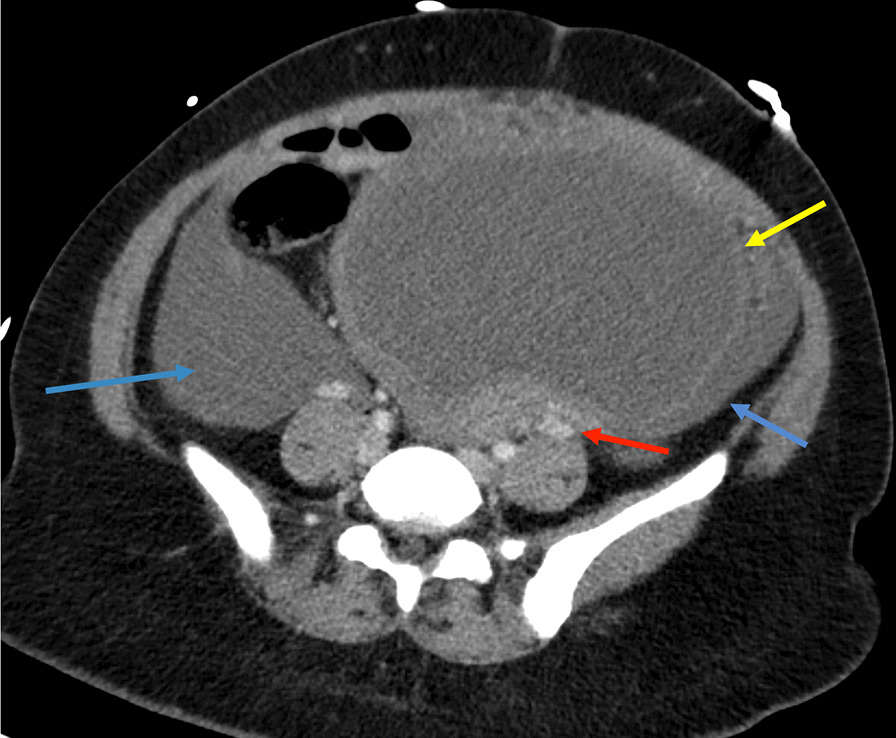
Large left pelvic hypodense structure (yellow arrow) drained by left gonadal vein (red arrow). There is moderate-volume ascites (blue arrows)

**Fig. 2:**
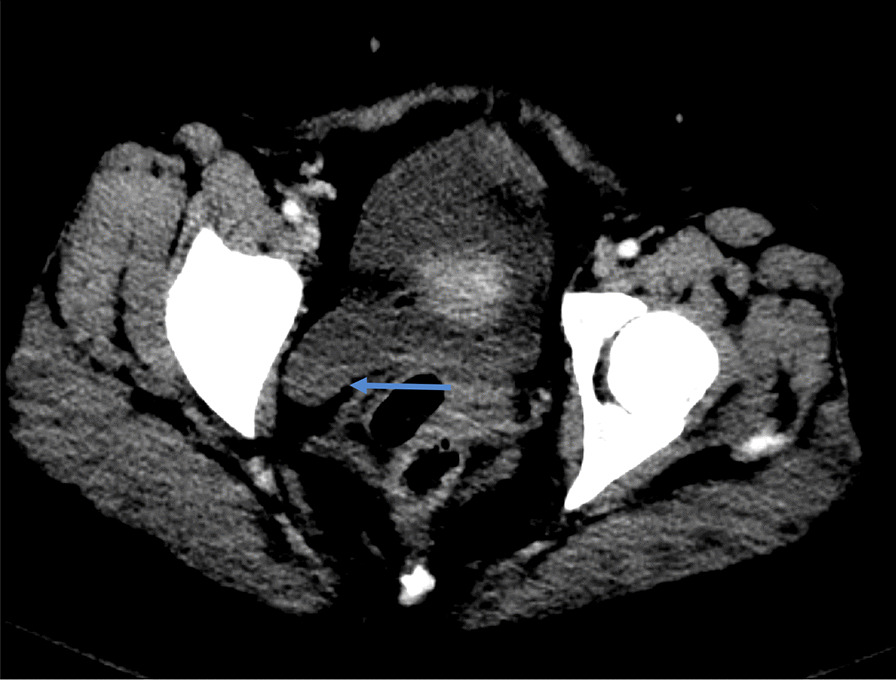
Hemoperitonieum with layering hyperdense material (blue arrow) compatible with blood products

**Fig. 3:**
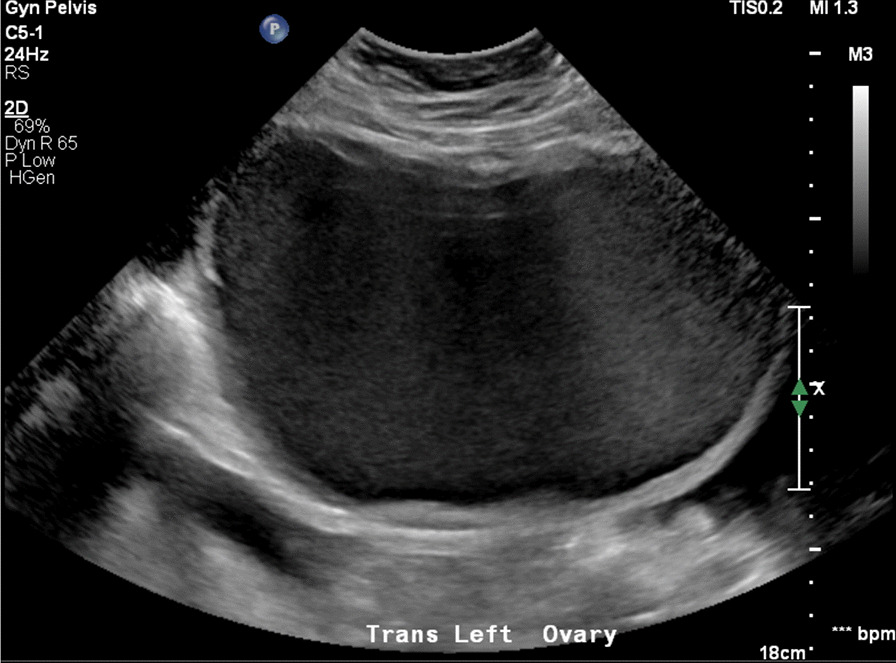
Large left ovarian cystic structure with homogeneous low-level internal echoes

**Fig. 4:**
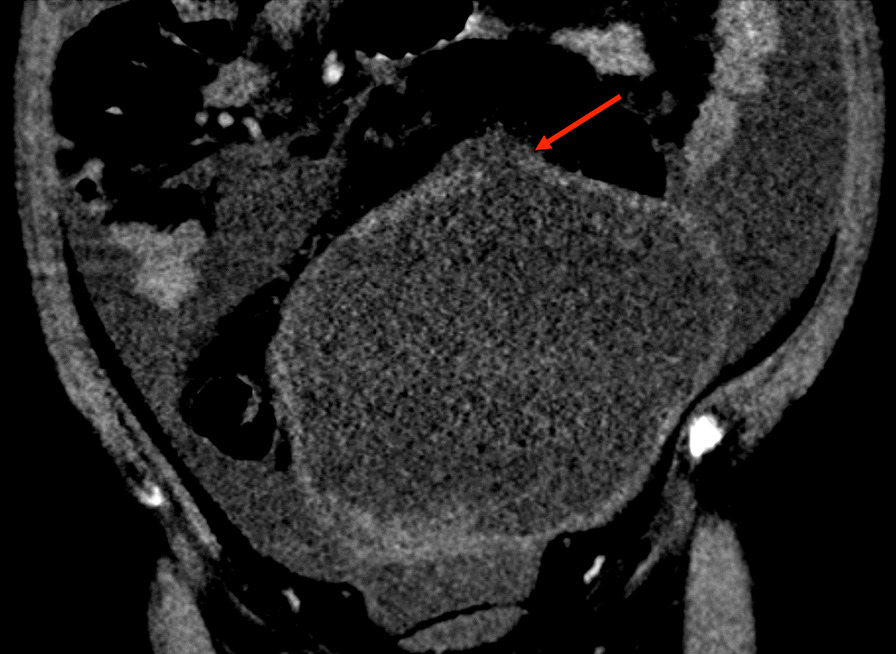
Focal rim discontinuity of the dome of the left adnexal cystic mass (red arrow)

Contrast-enhanced computed tomography (CT) of the abdomen and pelvis demonstrated a 13-cm complex cystic mass in the left adnexa with moderate hyperdense ascites and omental nodularity, suspicious for ovarian neoplasm with carcinomatosis (Figs. 1, 2, 3). Pelvic ultrasound demonstrated a large left adnexal complex cystic structure with internal echoes (Fig. 4). Chest CT did not reveal any intrathoracic neoplastic or infectious process. On diagnostic and therapeutic paracentesis, 700 mL of brownish opaque fluid was drained and sent for culture and cytology, which later revealed benign histocytes and mesothelial cells.

Subsequently, the patient underwent a diagnostic laparoscopy and tissue sampling by gynecologic oncology. The left ovary was found to be completely replaced by an endometrioma with a thickened outer rind deeply adherent to the pelvic wall. The cyst had a small ruptured area superiorly. Brown deposits of endometriosis were present on the cyst as well as the omentum and various peritoneal compartments sampled. Biopsy of the anterior abdominal wall was obtained, which later revealed a minute fragment of fibrous tissue with few pigmented macrophages and benign mesothelial cells.

**Fig. 5:**
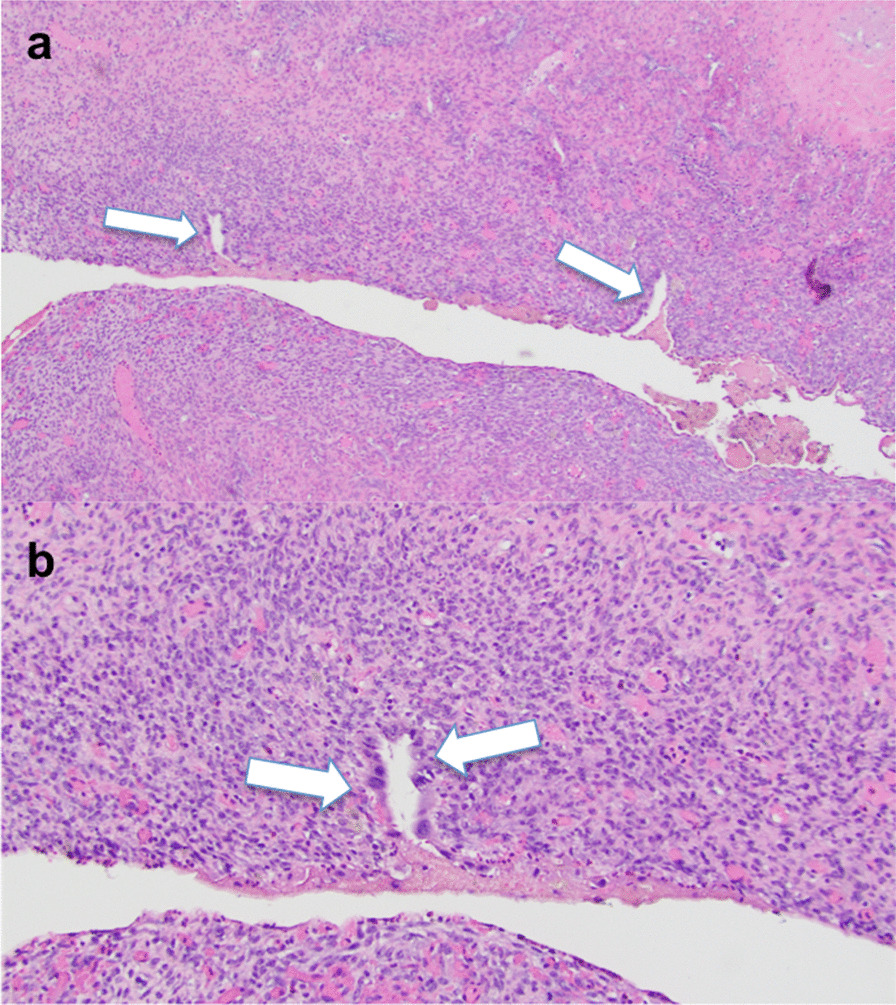
Hematoxylin and eosin stain of left ovary shows ovarian stroma with endometrial glands (white arrows) at 40× magnification (**a**) and 100× magnification (**b**)

Given that this patient had completed childbearing, she elected to undergo a modified radical hysterectomy with bilateral salpingo-oophorectomy, omentectomy, and peritoneal biopsies. Pathologic evaluation of the endometrium, myometrium, cervix, ovaries, fallopian tubes, peritoneum, omentum, and paracolic spaces demonstrated fibromuscular tissue with hemosiderin-laden macrophages and chronic inflammation suggestive of multiple foci of endometriosis (Fig. 5). No hyperplastic, dysplastic, or malignant cells were identified from any of the samples.

## Discussion

In this patient with a painful adnexal mass, recent weight loss, elevated CA-125, and ascites suspicious for carcinomatosis, malignancy was initially considered as the most likely diagnosis. However, it is important for clinicians and radiologists to consider the possibility of a ruptured endometrioma in patients with these findings. A ruptured endometrioma can cause marked elevation of CA-125 as well as cancer antigen 19-9 (CA 19-9) and d-dimer [5].

Imaging can be helpful in distinguishing between malignancy and endometrioma. On ultrasound, an endometrioma will typically appear as a cystic structure with low-level internal echoes [6]. This is especially true in premenopausal women, while endometriomas in postmenopausal women tend to deviate from this appearance on ultrasound [6]. Additionally, magnetic resonance imaging can be helpful in the assessment of endometriomas, as it can discern characteristics of blood products of varying ages—a hallmark of endometriomas [7]. When evaluating for rupture, it is important to recognize that a ruptured endometrioma or hemorrhagic cyst may present as an atraumatic complex adnexal cyst with large-volume hemoperitoneum. Features such as partial rim discontinuity of the adnexal cystic mass and hyperdense ascites further support the diagnosis of a ruptured endometrioma with hemoperitoneum. In contrast, imaging findings that are more suggestive of malignancy include thick enhancing septa, a centrally necrotic soft-tissue component, and papillary projections, which are generally absent in benign ovarian masses [8].

Despite these distinct imaging findings, laparoscopy is considered the gold standard for diagnosing endometriosis and endometrial cysts [7]. Especially in those patients without prior diagnosis of endometriosis, laparoscopy must be performed to confirm diagnosis, and biopsy must be performed to rule out malignancy.

## Conclusion

When premenopausal women present with hemoperitoneum in combination with complex adnexal cystic masses in the absence of trauma, differential diagnoses should include ruptured endometrioma and ruptured hemorrhagic cyst. CA-125 and CA 19-9 can be falsely elevated in the setting of ruptured endometrioma.

## Data Availability

Not applicable.

## Abbreviations

AST: Aspartate aminotransferase
ALT: Alanine transaminase
CA-125: Cancer antigen-125
CT: Computed tomography
CA19-9: Cancer antigen 19-9

## References

[1] Hoyle AT, Puckett Y. Endometrioma. In: StatPearls. Treasure Island (FL): StatPearls Publishing; 2020. https://www.ncbi.nlm.nih.gov/books/NBK559230/. Updated 12 June 2020.

[2] Evangelinakis N, Grammatikakis I, Salamalekis G, *et al*. Prevalence of acute hemoperitoneum in patients with endometriotic ovarian cysts: a 7-year retrospective study. Clin Exp Obstet Gynecol. 2009;36:254–5. 20101861

[3] Fonseca EK, Bastos BB, Yamauchi FI, Baroni RH. Ruptured endometrioma: main imaging findings. Radiol Bras. 2018;51(6):411–2. 10.1590/0100-3984.2017.0092 PMC6290756 30559563

[4] Pratt JH, Shamblin WR. Spontaneous rupture of endometrial cysts of the ovary presenting as an acute abdominal emergency. Am J Obstet Gynecol. 1970;108(1):56–62. 10.1016/0002-9378(70)90204-8 5465884

[5] Kurata H, Sasaki M, Kase H, Yamamoto Y, Aoki Y, Tanaka K. Elevated serum CA125 and CA19-9 due to the spontaneous rupture of ovarian endometrioma. Eur J Obstet Gynecol Reprod Biol. 2002;105:75–6. 10.1016/s0301-2115(02)00139-2 12270571

[6] Van Holsbeke C, Van Calster B, Guerriero S, Savelli L, Paladini D, Lissoni AA, Czekierdowski A, Fischerova D, Zhang J, Mestdagh G, Testa AC, Bourne T, Valentin L, Timmerman D. Endometriomas: their ultrasound characteristics. Ultrasound Obstet Gynecol. 2010;35(6):730–40. 10.1002/uog.7668. 20503240

[7] Glastonbury CM. The shading sign. Radiology. 2002;224(1):199–201. 10.1148/radiol.2241010361 12091683

[8] Jung SE, Lee JM, Rha SE, Byun JY, Jung JI, Hahn ST. CT and MR imaging of ovarian tumors with emphasis on differential diagnosis. Radiographics. 2002;22(6):1305–25. 10.1148/rg.226025033 12432104

